# The efficacy and safety of catheter balloon dilatation in the treatment of dysphagia after stroke: A protocol for systematic review and meta-analysis

**DOI:** 10.1097/MD.0000000000031460

**Published:** 2022-11-04

**Authors:** Lin Wang, Jian Yao, Bugao Guan, Jin Xu, Haitao Yu, Hongbo Li

**Affiliations:** a Rehabilitation Medicine Department, Jinhu County People’s Hospital, Huai’an City, Jiangsu Province, China.

**Keywords:** achalasia of the cricopharyngeal muscle, catheter balloon dilatation, dysphagia, protocol

## Abstract

Dysphagia is a serious complication after stroke, which has a significant influence on the health as well as life quality of global people. Patients with dysphagia tend to be a higher risk rate of an aspiration than general person. Catheter balloon dilatation is an additional therapy for treating dysphagia in recent years, which can improve the symptom of achalasia of cricopharyngeal muscle. This research will be used for confirming the efficacy and safety of the catheter balloon dilatation for resolving dysphagia.

**Methods:**

We intend to search literature related to the research in different databases, for instance, China National Knowledge Infrastructure, Wanfang Data, PubMed, Cochrane Library, and Embase up to January 2022. Literature selection, data collection, as well as assessment of bias risk, will be carried out by 2 independent researchers. Data analysis will be conducted by using Stata and review manager 5.4.

**Results:**

The results will be submitted to a peer-reviewed journal.

**Conclusion:**

The research will verify whether or not catheter balloon dilatation can improve dysphagia by submitting high-quality data syntheses.

**Registration number:**

CRD42022358433.

## 1. Introduction

Stroke is the leading cause of disability and a major cause of mortality worldwide,^[1]^ which has a greater effect on women than men.^[2]^

Dysphagia is one of the most common post-stroke sequelae, accounting for 27% to 64% of stroke patients,^[3]^ with a prevalence of 10% remaining after 6 months.^[4]^

Swallowing is a complex process that concerns the coordination of multiple muscle groups to push the food from the mouth to the stomach while protecting the airway and minimizing residue. The neural circuit that produces the swallowing pattern is located in the medulla oblongata.^[5]^

Risk factors for dysphagia include Older age,^[6,7]^ Greater NIHSS,^[6–11]^ Lower Barthel index,^[12,13]^ Malnutrition or lower body mass index at admission,^[12,14,15]^ Greater lesion volume,^[10,11,16,17]^ Subcortical or cortical involvement,^[18–20]^ Brainstem involvement,^[9,18,19,21–24]^ Corticobulbar tract involvement,^[13,25]^ White matter involvement,^[9,13,25]^ Presence of dysarthria,^[16,22]^ Presence of dysphonia or reduced maximum pitch,^[16,26]^ and Cognitive impairment or dementia.^[27,28]^

The consequences of dysphagia after a stroke include an increased risk of pulmonary complications, dehydration, malnutrition, and death.^[29,30]^

Treatment for dysphagia after a stroke includes several approaches that exist for treating cricopharyngeal dysfunction, including swallowing rehabilitation, cricopharyngeal balloon dilation, botulinum toxin injection, and cricopharyngeal myotomy.^[31]^

Therefore, it is necessary to find a therapy with high safety and convenience to improve dysphagia for stroke patients. Balloon dilatation is a technique used to treat pharyngeal dysphagia, balloon dilation for neurogenic dysphagia has been widely used in clinical practice, and its usefulness has been supported,^[32]^ in 1 systematic review, the success rate of dilation was between 58% and 100% (mean = 81%).^[31]^

Recently, there have been many studies on correcting patients’ dysphagia by balloon dilatation, which have some differences among different studies. The aim of the meta-analysis is to evaluate how well and safely rope therapy helps people who have trouble with their balance after a stroke.

## 2. Study registration

This study has been registered on the PROSPERO. The registration number is CRD42022358433. The method used for this protocol will follow Preferred reporting items for systematic review and meta-analysis protocols statement.

### 2.1. Types of studies

The types of included studies are randomized controlled trials of the efficacy and safety of catheter balloon dilatation in the treatment of dysphagia caused by achalasia of the cricopharyngeal muscle after stroke. We will exclude them if the types of articles are cases, reviews, abstracts, and comments.

### 2.2. Types of participants

Patients must fulfill nationally or globally accepted diagnostic standards for post-stroke dysphagia, such as those outlined in the European Society for Swallowing Disorders’ White Paper. The minimum patient age is 18, and there are no restrictions on the patient’s gender, race, or illness course. Patients with dysphagia brought on by other illnesses are excluded.

### 2.3. Types of interventions

We will consider mainly using catheter balloon dilatation as the experimental group.

### 2.4. Types of comparators

We will consider other treatments as the control group, except catheter balloon dilatation.

### 2.5. Types of outcome measures

We will consider the following methods as the outcome measures: efficacy, video fluoroscopic swallowing study score, Water Swallowing Test, Standard Swallowing Assessment Scale score, Symptom score criteria, and Functional Oral Intake Scale.

We will take clinical efficacy as the primary outcome. Swallowing function will be considered as a secondary outcome.

### 2.6. Exclusion criteria

[1] Repeated published literature.
[2] Analysis of missing data.
[3] Retrospective literature.
[4] Non-Chinese and English literature.
[5] Literature with no needed outcomes in the literature.
[6] Literature with unqualified trial design (e.g., no explicit trial control in the trial).
[7] Non-randomized controlled trials
[8] Animal trials.

### 2.7. Information sources

We will search the China National Knowledge Infrastructure, WanFang Data, Chinese biological medical database, Cochrane Library, and PubMed databases for the literature concerning the efficacy and safety of catheter balloon dilatation in the treatment of dysphagia after a stroke up to January 1, 2022.

### 2.8. Search strategy

We will search several databases for relevant material. The following keywords will be included in the search terms: randomized controlled trial, stroke, dysphagia, catheter balloon dilatation, and their respective Medical Subject Headings.

We will also look for any relevant current or unpublished studies on the WHO International Clinical Trial Registry Platform, the Chinese Clinical Trial Registry, ClinicalTrials.gov, and Google scholar.

The Search strategy will be offered to peer-review is Table 1.

**Table 1:** The search strategy was used in the Pubmed database.

Search number	Search terms
1	Randomized controlled trial
2	controlled clinical trials
3	Randomly
4	Randomized
5	Trial
6	Cerebrovascular accident
7	CVA
8	Cerebrovascular apoplexy
9	Vascular accident, Brain
10	Cerebrovascular stroke
11	Cerebral stroke
12	Acute stroke
13	Acute cerebrovascular accident
14	Stroke
15	Deglutition disorders
16	Swallowing disorders
17	Dysphagia
18	Oropharyngeal dysphagia
19	Esophageal dysphagia
20	Balloon dilation
21	1 OR 2–5
22	8 OR 9–16
23	17 OR 18–21
24	22 AND 23–25

CVA = cerebrovascular accident.

### 2.9. Data management

Two researchers will extract essential information and statistical data from the listed papers separately. Endnote X9 will be used to maintain suitable literature, and Microsoft Excel will be used to create forms for basic information and study statistic data.

### 2.10. Selection process

Two researchers will be assigned to independently identify qualifying studies. For preliminary screening, we first searched the databases for relevant literature by reading the title and abstract. Then, we will download and read the whole text of the remaining literature. Finally, after removing all of the literature using exclusion criteria, the remaining literature will be included in the systematic review and meta-analysis. Assuming that there is a discrepancy between the 2 researchers’ selections of literature, the third researcher will enter the conversation and settle it.

### 2.11. Data collection process

The data gathering process will be carried out by 2 researchers. To begin, we will gather qualifying information in Microsoft Excel. In the event of disagreements, the third researcher will take part in the debate and solution. The data will be extracted using a pre-designed data extraction table, which will include the following information: the basic information included in the study (the first author’s name, the sample size, and the publication date); the essential characteristics of the subjects (the age and gender of the participants); the specific details of the intervention and control groups; the cycle of the trial; the duration; the outcome indicators; adverse events; and the frequency of a single intervention.

If the original study data description in the literature is unclear or there is a paucity of data, we will contact the author through email or phone to finish it.

### 2.12. Study risk of bias assessment

Two independent researchers will conduct the risk of bias assessment. The quality evaluation criteria recommended by Cochrane Review^[18]^ include random sequence generation (selection bias), allocation concealment (selection bias), blinding of participants and personnel (performance bias), blinding of outcome assessment (detection bias), incomplete outcome data (attrition bias), selective reporting (reporting bias), and other bias.

We will make judgments of “yes” (low risk), “no” (high risk), and “unclear” (lack of relevant information or uncertainty of bias) for each project.

### 2.13. Statistical analysis

Two researchers will conduct a meta-analysis by Review Manager 5.4 independently. The combined mean difference with 95% confidence intervals (CI) will be used for the continuous data and the combined odds ratio and relative risk with 95% CI will be used for the dichotomous data.

The Chi-square test will be used to evaluate the statistical heterogeneity among the results of the studies. The size of heterogeneity will be quantitatively judged by *I*^*2*^. When the test results are *P* > .05 and *I*^*2*^ < 50%, the fixed effect model will be used for meta-analysis. If the test results are *P* < .05 and *I*^*2*^ > 50%, we will analyze the recourses of the heterogeneity and choose the random effect model.

Subgroup analyses will be carried out on the assumption that the heterogeneity is significant. If there is an outcome that includes 10 or more studies, we will make a funnel chart for publication bias assessment.

### 2.14. Confidence in cumulative evidence

We will use the Grades of Recommendations Assessment Development and Evaluation approach to verify the quality of evidence of the included studies. The Grades of Recommendations Assessment Development and Evaluation approach divides the quality of evidence into very low, low, moderate, or high.

## 3. Ethics approval

The study does not require ethical approval because of failing to involve the patients.

## 4. Discussion

The research aims to verify the efficacy and safety of catheter balloon dilatation in the treatment of dysphagia after stroke for clinical promotion. The catheter balloon dilatation is a prevailing method that is used increasingly for dysphagia in recent years. However, there is no meta-analysis concerning the safety and efficacy of catheter balloon dilatation. So, It is necessary to testify to the safety and efficacy of catheter balloon dilatation if it can be used as an alternative to the traditional treatment.

## Acknowledgments

The authors would like to express their gratitude to the Huai’an Commission of Health.

## Author contributions

**Conceptualization:** Hongbo Li.

**Data curation:** Yao Jian.

**Formal analysis:** Jin Xu, Haitao Yu.

**Funding acquisition:** Hongbo Li.

**Methodology:** Guanbu Gao, Lin Wang.

**Resources:** Yao Jian, Lin Wang.

**Software:** Guanbu Gao, Haitao Yu.

**Writing – original draft:** Yao Jian, Hongbo Li, and Jin Xu.

**Writing – review & editing:** Guanbu Gao, Lin Wang, and Haitao Yu.
